# Activity-Based Protein Profiling Reveals That Cephalosporins Selectively Active on Non-replicating *Mycobacterium tuberculosis* Bind Multiple Protein Families and Spare Peptidoglycan Transpeptidases

**DOI:** 10.3389/fmicb.2020.01248

**Published:** 2020-06-23

**Authors:** Landys Lopez Quezada, Robert Smith, Tania J. Lupoli, Zainab Edoo, Xiaojun Li, Ben Gold, Julia Roberts, Yan Ling, Sae Woong Park, Quyen Nguyen, Frank J. Schoenen, Kelin Li, Jean-Emmanuel Hugonnet, Michel Arthur, James C. Sacchettini, Carl Nathan, Jeffrey Aubé

**Affiliations:** ^1^Department of Microbiology & Immunology, Weill Cornell Medical College, New York, NY, United States; ^2^Chemical Methodologies & Library Development Center, The University of Kansas, Lawrence, KS, United States; ^3^Sorbonne Université, Sorbonne Paris Cité, Université de Paris, INSERM, Centre de Recherche des Cordeliers, CRC, Paris, France; ^4^Departments of Biochemistry and Biophysics, Texas A&M University, College Station, TX, United States; ^5^Division of Chemical Biology and Medicinal Chemistry, UNC Eshelman School of Pharmacy, The University of North Carolina at Chapel Hill, Chapel Hill, NC, United States

**Keywords:** *M. tuberculosis*, cephalosporin, non-replicating, β-lactams, ABPP, click chemistry

## Abstract

As β-lactams are reconsidered for the treatment of tuberculosis (TB), their targets are assumed to be peptidoglycan transpeptidases, as verified by adduct formation and kinetic inhibition of *Mycobacterium tuberculosis* (Mtb) transpeptidases by carbapenems active against replicating Mtb. Here, we investigated the targets of recently described cephalosporins that are selectively active against non-replicating (NR) Mtb. NR-active cephalosporins failed to inhibit recombinant Mtb transpeptidases. Accordingly, we used alkyne analogs of NR-active cephalosporins to pull down potential targets through unbiased activity-based protein profiling and identified over 30 protein binders. None was a transpeptidase. Several of the target candidates are plausibly related to Mtb’s survival in an NR state. However, biochemical tests and studies of loss of function mutants did not identify a unique target that accounts for the bactericidal activity of these beta-lactams against NR Mtb. Instead, NR-active cephalosporins appear to kill Mtb by collective action on multiple targets. These results highlight the ability of these β-lactams to target diverse classes of proteins.

## Introduction

The chemotherapy of tuberculosis (TB) remains challenging, with an urgent need for shorter, safer treatment whose effectiveness extends to TB resistant to current regimens (Murray et al., 2016). Cure of a high proportion of patients with drug-sensitive TB depends on use of multiple antibiotics for at least 6 months. In part, this is thought to reflect the phenotypic antimicrobial resistance that is characteristic of non-replicating (NR) bacterial populations (Nathan, 2012). To this end, several strategies have been proposed to improve the treatment course against NR bacteria (Murray et al., 2016; Gold and Nathan, 2017). One strategy involved a search for drugs that can kill Mtb *in vitro* when it has been made NR by conditions that mimic those found in the host (Bryk et al., 2008; Mak et al., 2012; Grant et al., 2013; Gold and Nathan, 2017).

The bacterial cell wall is the target of many antibiotics, β-lactams among them. However, the cell wall of mycobacteria was considered refractory to inhibition by β-lactams for decades following the demonstration that penicillin was inactive against Mtb (Abraham et al., 1941). β-Lactams are the most highly prescribed antibiotics in modern medicine (Bush and Macielag, 2010) and have utility in diverse infections (Wivagg et al., 2014). Mtb’s resistance to β-lactams was attributed to the natural resistance of its peptidoglycan transpeptidases, the robust production of a class A β-lactamase (BlaC), and the limited permeability of its thick, waxy outer cell wall. Peptidoglycan peptide crosslinking in mycobacteria relies largely but not completely on L,D-transpeptidases (Ldts) that catalyze transpeptidation via an active site cysteine rather than the serine of D,D-transpeptidases (Mainardi et al., 2005). D,D-transpeptidases along with D,D-carboxypeptidases and D,D-endopeptidases are the classical targets of β-lactams and are collectively called penicillin binding proteins (PBPs). In Mtb, as much as 80% of peptidoglycan crosslinks are mediated by Ldts (Lavollay et al., 2008) when cells are in stationary phase compared to 30–40% of the crosslinks in replicating cells (Wietzerbin et al., 1974).

Over the years, interest in β-lactams for the treatment of TB has undergone a renaissance, spurred in part by the discovery that the carbapenem, meropenem, combined with the β-lactamase inhibitor clavulanic acid, sterilized replicating cultures of Mtb within 2 weeks of incubation (Hugonnet et al., 2009), along with the demonstration of the vulnerability of Ldts to β-lactam inhibition. A long-term case study of 18 patients with extensively drug-resistant TB concluded that including meropenem/clavulanate in the treatment regimen was beneficial to outcomes (Payen et al., 2018). While various carbapenems, including faropenem, are the most effective β-lactams at inhibiting Mtb Ldts (Kumar et al., 2017), cephalosporins also bind the Ldts of Mtb (Dubee et al., 2012b; Kumar et al., 2017) and *Enterococcus faecium* (Dubee et al., 2012a). Also, a previous study observed that the activity of rifampicin, a first-line antimycobacterial, is enhanced when co-administered with several cephalosporins *in vitro* (Ramón-García et al., 2016). The underlying basis of this synergy remains unknown.

We recently screened a β-lactam library against Mtb under both replicating and NR conditions that mimic the host environment, the latter set of conditions consisted of hypoxia, low pH, a flux of nitric oxide generated from nitrite, and butyrate as a carbon source (Gold et al., 2015b). We found two cephalosporins, compounds **1** and **5**, that were exclusively active against NR Mtb and were cidal to Mtb in mouse bone marrow-derived macrophages (Gold et al., 2016b). Restriction of activity to NR Mtb raised the question whether these cephalosporins might have non-canonical targets (Baranowski and Rubin, 2016) beyond transpeptidation enzymes.

Further suggesting the likelihood of non-canonical targets, the NR-active cephalosporins bear an ester or an oxadiazole (an ester isostere) at the C2′ position of the cephalosporin core, rather than the free carboxylic acid characteristic of inhibitory cephalosporins (Chauvette and Flynn, 1966; Jen et al., 1972). The importance of the free carboxylic acid is highlighted in the crystal structure of the acyl-enzyme tetrahedral intermediate between PBP2x from *Streptococcus pneumoniae* and cefuroxime, which showed tight hydrogen bonding between Thr550 in the active site and the carboxylic acid of cefuroxime (Gordon et al., 2000). Loss of this interaction with Thr550 reduced affinity for 2nd- and 3rd-generation cephalosporins and increased resistance to cephalosporins in clinical isolates (Mouz et al., 1999). Furthermore, the crystal structures of the Mtb Ldt_Mt__1_ (Correale et al., 2013) and Ldt_Mt__2_ (Bianchet et al., 2017) showed tight hydrogen bonding interactions between three active site side chains and the carboxylate of several carbapenems bound to the active site cysteine.

Alternative targets of β-lactams, with no obvious relationship to enzymes involved in peptidoglycan metabolism, have been reported in other organisms. A monobactam inhibited the *Escherichia coli* signal peptidase SPase (Kuo et al., 1994). Crystallization of *E. coli* SPase with a β-lactam was achieved using a penem with an ester at the carboxylate position (Paetzel et al., 1998). Certain cephalosporins esterified at the C2′′ carboxylate inhibit human leukocyte elastase (HLE) (Doherty et al., 1986).

In the NR state, peptidoglycan biosynthesis is anticipated to slow or completely arrest as the bacilli halt replication. Ldts, obvious candidate targets of β-lactams, play an essential role for replicating Mtb and their role in maintaining peptidoglycan synthesis in NR Mtb is not as well understood. Thus, we first tested if NR-active cephalosporins inhibited recombinant Mtb Ldts. To explore the possibility of non-canonical targets, we then turned to activity-based protein profiling (ABPP) to seek other potential target(s).

ABPP has been adopted to find binding partners for ligands that target cysteine, serine, and metallohydrolases, ATP-binding proteins, phosphatases, and other enzyme classes (Cravatt et al., 2008). ABPP makes use of alkyne or azide analogs of the ligand of interest, which can then be conjugated via a Cu(I)-mediated azide alkyne cycloaddition reaction to create a handle for enrichment and purification or labeling (Speers and Cravatt, 2009), followed by peptide mass fingerprinting to identify binding proteins. In Mtb, ABPP has been used to elucidate the serine hydrolase landscape of replicating and hypoxic NR cells (Ortega et al., 2016) and to identify a protein that binds agrimophol, a cidal natural product that disrupts Mtb’s intrabacterial pH homeostasis (Zhao et al., 2015).

In this study, by using ABPP, we identified candidate target proteins for NR-active cephalosporins that belonged to diverse protein families. Conspicuously absent were Ldts and PBP transpeptidases and carboxypeptidases.

## Results

### NR-Active Cephalosporins Do Not Inhibit Mtb Ldts and Are Not Susceptible to BlaC Cleavage

**FIGURE 1:**
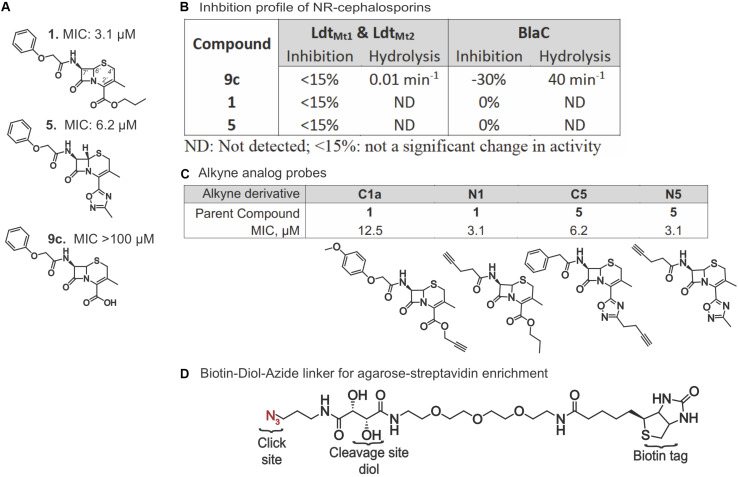
Activity and design of alkyne probes for ABPP. **(A)** Structures and non-replicating (NR) MICs of compounds **1**, **5**, and **9c**. **(B)** Percent inhibition of nitrocefin hydrolysis and compound hydrolytic activity of Ldt_Mt1_, Ldt_Mt2_, and BlaC when incubated with compounds **1**, **5**, and **9c**. **(C)** Structure and NR MICs of alkyne derivatives. **(D)** Structure of the biotin-diol linker used for purification and identification of proteins bound to the cephalosporin probes.

Given the importance of Ldts in Mtb’s adaptation to stress conditions, we first queried if there was any notable interaction between Mtb Ldts and compounds **1**, **5**, and the carboxylic acid analog, **9c** (Figure 1A). Ldt_Mt__1_ and Ldt_Mt__2_ were selected for their critical role in mycobacterial biology (Schoonmaker et al., 2014). No inhibitory activity was detected (Figure 1B) with or without prior incubation with the compounds. The molar extinction coefficient (Δε) of the hydrolyzed compounds was determined by incubating **1**, **5**, and **9c** with *Klebsiella pneumoniae* carbapenemase, KPC-2. Partial hydrolysis of **1** and **5** was achieved after 1,000 min while **9c** was rapidly hydrolyzed. Neither compound **1** nor **5** could be hydrolyzed by the Ldts. Furthermore, stopped-flow kinetics were performed using *E. faecium*. Ldt_fm_ failed to detect an interaction with **1** or **5**. Considering that **9c** interacted with Ldt_Mt__1_, Ldt_Mt__2_, and Ldt_fm_ as expected, it seemed likely that the lack of a charged carboxylate alters the binding of the compounds and, most likely, **1** and **5** do not have canonical cephalosporin targets. Next, we tested the susceptibility of the three compounds to hydrolysis by Mtb BlaC. Mtb BlaC was unable to hydrolyze **1** or **5**; however, as with KPC-2, **9c** was readily degraded with a turnover rate of 40 min^–1^ (Figure 1B). Neither **1** nor **5** inhibited BlaC hydrolysis of nitrocefin, even after a 2-h pre-incubation. These findings led us to opt for an unbiased approach to finding potential targets of **1** and **5**.

### Chemo-Proteomic Approach to Target Identification

**FIGURE 2:**
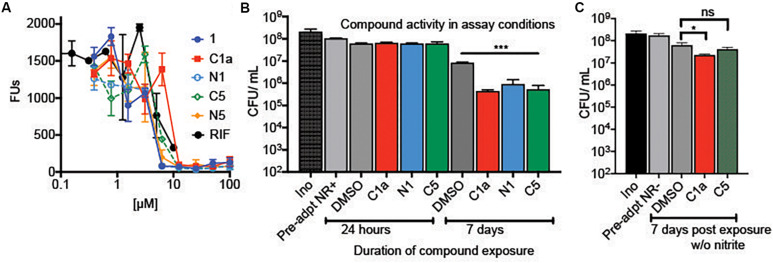
Activity of alkyne analogs against Mtb at a high inoculum. **(A)** Activity of compound **1** and alkyne derivatives against NR Mtb in standard NR conditions at an initial OD_580_ of 0.1. **(B)** Cidality of alkyne analogs in the non-replicating pull-down assay conditions that included a 24-h pre-exposure to NR conditions and a high inoculum (OD_580_ of 1.0). Cells were exposed to alkyne analogs for 24 h or 7 days. After 7 days of exposure, **C1a**, **N1**, and **C5** significantly reduced viability (***p < 0.005). **(C)** Activity of alkyne analogs after 7 days under NR conditions without the flux of nitric oxide, *p = 0.045.

We synthesized alkyne analogs of **1** and **5** that retained NR activity comparable to that of the parent compounds (Figures 1A,C). The alkyne tag was coupled to either the C2′ carboxyl (C) or C7′ amino (N) group of each cephalosporin, yielding compounds **C1a**, **N1**, **C5**, and **N5**. All four tagged compounds retained NR activity and none gained activity against replicating Mtb (replicating MIC90 ≥ 100 μM; data not shown). Thus, we were able to apply the probe compounds to intact, live NR Mtb rather than having to treat lysates prepared from NR Mtb. Achieving reproducible and compound-specific labeling of both membrane-associated and cytoplasmic proteins required overcoming several technical challenges. In our preliminary experiments, we found that use of a biotin azide or a desbiotin azide linker led to problems with the elution of non-specific proteins bound to the agarose-streptavidin resin and the contamination of samples with streptavidin, which could mask target proteins of low abundance. We remedied this with a biotin linker containing a 1,2-diol near the azide (Figure 1D) that allowed periodate-mediated oxidative cleavage of the linker. We used a high biomass of Mtb to increase the likelihood of capturing low abundance protein(s). This required setting up experiments using a high bacterial inoculum and adjusting the compound concentrations to account for the inoculum effect observed in **1**, **5**, and many other β-lactams (Gold et al., 2016b), in which the compounds became less potent as the starting inoculum was raised from 0.01 OD_580_ to 0.1. Not only did the antimycobacterial activity of the alkynes mirror the activity of the parent compounds in standard MIC and charcoal agar resazurin assays (CARA) (Figures 1C, 2A) where the OD_580_ is adjusted to 0.01, but they remained cidal despite the high inoculum of 5 × 10^8^ CFU/ml (an approximate OD_580_ of 1.0) after 7 days of incubation (Figure 2B). Similar to the parent compounds (Gold et al., 2016b), activity of their alkyne analogs was largely dependent on the inclusion of nitrite in the NR conditions (Figure 2C). The standard MIC of the alkyne analogs and the parent compounds was comparable to that of oxyphenbutazone (OPB, Supplementary Table S1), which is active in the non-replicating 4-stress model (Gold et al., 2012).

**FIGURE 3:**
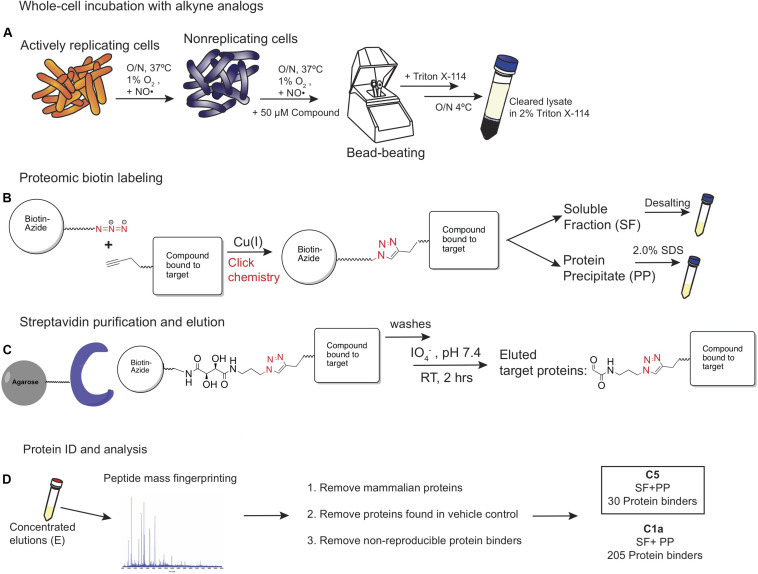
The ABPP strategy used for labeling potential protein binders to **C1a**, **N1**, **C1**, and **N5**. **(A)** The strategy involved 24-h exposure of NR pre-conditioned Mtb cells to test compounds, lysis, and solubilization of cellular and membrane proteins in Triton X-114. **(B)** Conjugation of the probe–protein complex to the diol-biotin linker via a Cu(I)-catalyzed Huisgen’s azide-alkyne cycloaddition reaction. **(C)** Streptavidin purification followed by periodate/diol linker cleavage. **(D)** Mass spectrometry analysis of eluents and removal of non-specific protein binders.

**FIGURE 4:**
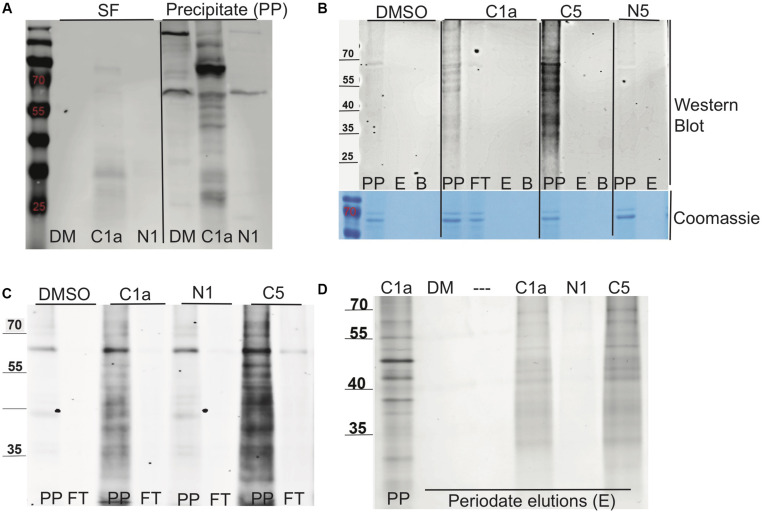
Biotin labeling, streptavidin purification, and elution of proteins bound to NR-active cephalosporins. **(A)** The labeling of lysate proteins with click chemistry reaction was visualized by Western blot using Alexa Fluor streptavidin conjugate (Thermo Fisher Scientific, United States). The soluble fraction (SF) or precipitated (PP) protein samples treated for 24 h with DMSO (DM), **C1a**, or **N1**. **(B)** Western blot of the PP fraction following streptavidin purification of samples treated with DMSO, **C1a**, **C5**, or **N5**; precipitate (PP), periodate elution (E), boiled bead elution (B), streptavidin flow through (FT). Directly underneath is a loading control protein gel stained with Coomassie. **(C)** Western blot of PP fraction binding to streptavidin beads of samples treated with DMSO, **C1a**; **N1**; **C5**; precipitate (PP), streptavidin flow through (FT). **(D)** Silver staining of periodate elution (E) and **C1a** precipitate for comparison.

The methods used to label, enrich, purify, and elute bound target proteins are shown schematically in Figure 3. **C1a**, which contained the alkyne handle at the C2′ carboxylic acid group, labeled the proteome more robustly than the **N1** probe, which carried the alkyne at C7′ amino moiety (Figure 4A). The same pattern was observed between **C5** and **N5** probes (Figure 4B), suggesting preferential orientation of these cephalosporins with binding partners to which they could most readily undergo the azide alkyne cycloaddition reaction and subsequent detection. Boiling the beads after the oxidation reaction did not release any additional biotinylated proteins (Figure 4B), suggesting that the periodate chemical cleavage was complete. Furthermore, no proteins were detected in the flow-through (Figure 4C), which indicated that binding of the tagged proteins to the streptavidin-agarose was comprehensive. Having removed the biotin tag, we visualized the proteins in the concentrated eluates by silver staining the SDS-PAGE gels (Figure 4D).

### C5 and C1a Binding Proteins Are Not Involved in Peptidoglycan Synthesis

After optimizing assay conditions and purification steps (Figure 3), we conducted ABPP on two independent experiments using **C1a**, **C5**, and a vehicle control (1% DMSO). No probe-specific proteins were identified with the **N1** or **N5** probes and, therefore, analytical efforts were focused on results with **C1a** and **C5**. The **C5** probe pulled down a much smaller set of protein binders (39 proteins) than the **C1a** probe (426 proteins) (Table 1). The majority were not detected in the vehicle-treated control cells. Of the proteins identified using **C5**, 93% were also identified using **C1a**. In order to assess the specificity of binding, ABPP with **C1a** was conducted in the presence of **1** or **5** as competitors. Recovery of 185 of the 205 reproducible candidate binders was reduced by at least 100-fold when **C1a** was co-incubated with **1** and unaffected by co-incubation with **5** (Table 1 and Supplementary Table S2). The set of 185 proteins included 28 of the 30 proteins that were pulled down with **C5**. Thus, the 30 proteins identified with **C5** were considered to be the most reproducible and specifically targeted by the NR-active cephalosporins. These were chosen for further study.

**TABLE 1:** Selection criterion and total number of final proteins identified.

Parent compound	**1**	**5**
Alkyne derivative (bait)	**C1a**	**C5**
Bacterial proteins with >2 unique peptides	426	39
Not found in DMSO	97%	82%
Competed with parent compound	45%	Not determined
Reproducible binders	48%	**77%**

	**% of total number of proteins ID’d**	

**Precipitate fraction**		
Bacterial proteins with >2 unique peptides	413	29
Not found in DMSO	406	24
Reproducible binders	**197**	**22**
***Soluble Fraction***		
Bacterial proteins with >2 unique peptides	13	10
Not found in DMSO	**8**	**8**

*Numbers used to tabulate pull down totals and are bolded for emphasis.*

### Genetic and Biochemical Studies of Non-essential Binding Protein

The proteins isolated with the **C5** probe belonged to several protein families, 16 of which are essential under replicating conditions (Table 2). Notably, known targets of β-lactams were not among them. Given that the compounds are specifically cidal to NR Mtb, we initially reasoned that these essential proteins may not be the targets whose inhibition would account for cidality only under NR conditions. Several of the proteins are predicted or have been verified to be involved in the following functions: fatty acid biosynthesis: DesA2, DesA1, HtdY, and AcpM; β-oxidation: EchA1, FadD31, and Rv3224; ribosome function: Tsf, Tuf, RplJ, and Rv1738; universal stress proteins: Rv2005, Rv2623, and Rv1996 (Table 2). Of the proteins with non-essential roles under replicating conditions, 13 of the 14 NR-cephalosporin-binding proteins that are not essential under replicating conditions (DeJesus et al., 2017) were assessed for their potential essentiality under NR conditions.

**TABLE 2:** Potential cephalosporin binding proteins from NR cells.

Accession	Rv number	Gene	Essentiality?	Description
**Fatty acid metabolism**				
L7N5P2	Rv3224		No	PP: oxidoreductase, short-chain dehydrogenase/reductase family
O53442	Rv1094	desA2	Yes	PP: putative acyl-[acyl-carrier-protein] desaturase
Q7D7S1	Rv1925	fadD31	No	PP: acyl-CoA synthase
Q50824	Rv0685	desA1	Yes	PP: putative acyl-[acyl-carrier-protein] desaturase
L7N5S5	Rv0222	echA1	No	SF: probable enoyl-CoA hydratase; crotonase
P0A4W6	Rv2244	AcpM	Yes	SF: meromycolate extension acyl carrier protein
Q11198	Rv3389c	htdY	No	SF: 3-hydroxy acyl thioester dehydratase
**Ribosomal/transcriptional function**				
P66044	Rv0651	rplJ	Yes	PP: 50S ribosomal protein L10
Q10788	Rv2889c	tsf	Yes	PP/SF: elongation factor Ts
P0A558	Rv0685	tuf	Yes	PP: elongation factor Tu
P66701	Rv3457c	rpoA	Yes	PP: DNA-directed RNA polymerase subunit alpha
P64887	Rv1738	–	No	SF: uncharacterized protein Rv1738; potential hibernation factor
**Universal stress proteins and chaperones**				
P0A5F7	Rv1996	Usp	No	PP: universal stress protein; appears redundant
P64411	Rv2299c	htpG	No	PP: chaperone protein
P64921	Rv2005c	Usp	No	PP: universal stress protein; appears redundant
O06189	Rv2623	Usp	No	PP: universal stress protein
**ATP synthesis**				
P63671	Rv1309	atpG	Yes	PP: ATP synthase gamma chain
P63673	Rv1308	atpA	Yes	PP: ATP synthase subunit alpha
P63677	Rv1310	atpD	Yes	PP: ATP synthase subunit beta
**Intermediary metabolism enzymes**				
P0A544	Rv2996c	serA	Yes	PP: D-3-phosphoglycerate dehydrogenase
P60176	Rv3248c	sahH	Yes	PP: adenosylhomocysteinase; thioester hydrolase
P64178	Rv1436	gap	Yes	PP: glyceraldehyde-3-phosphate dehydrogenase
P65149	Rv3001c	ilvC	Yes	PP: ketol-acid reductoisomerase
Q10530	Rv0896	gtlA2	Yes	PP: citrate synthase 1
P0A590	Rv2220	glnA1	Yes	PP: glutamine synthetase 1
P95143	Rv0694	lldD	No	PP: putative L-lactate dehydrogenase
P65682	Rv2607	pdxH	No	SF: pyridoxine/pyridoxamine 5′-phosphate oxidase
**Unknown function**				
O53291	Rv3044	fecB	No	SF: probable Fe(III)-dicitrate binding lipoprotein
O53672	Rv0250c	–	No	SF: uncharacterized protein
P71839	Rv0786c	–	No	SF: conserved protein

*C5 pulldown proteins: PP, precipitate fraction; SF, soluble fraction.*

**FIGURE 5:**
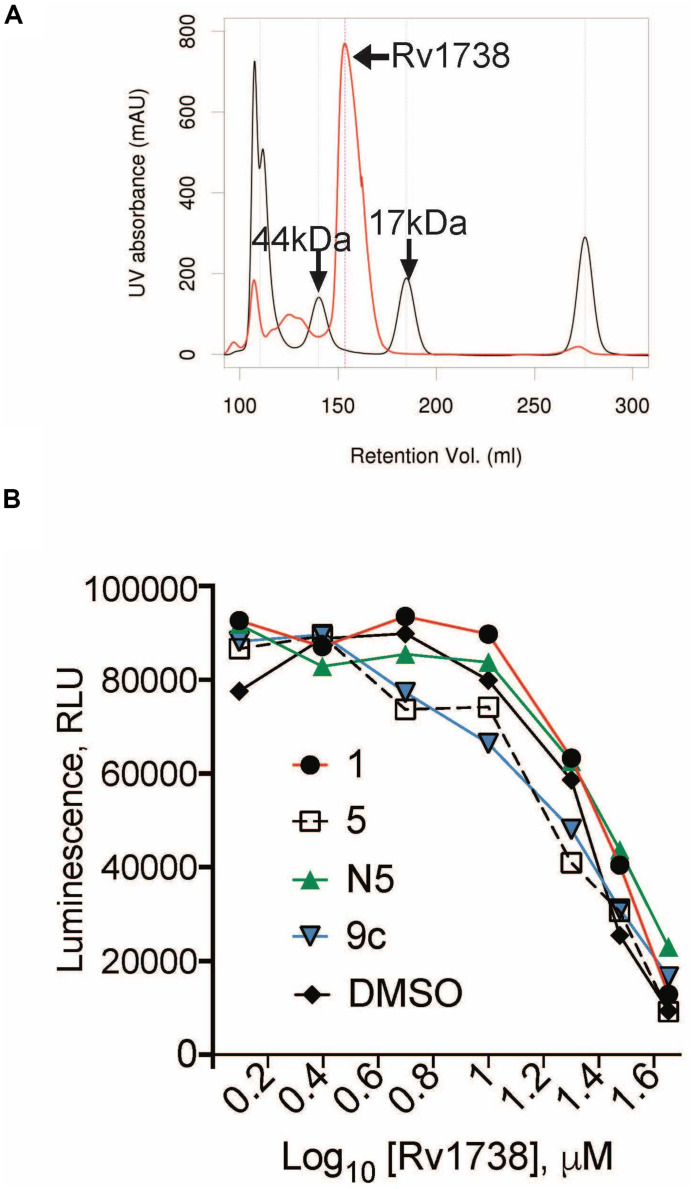
Characterization of Rv1738 as a potential hibernation factor. **(A)** Size exclusion chromatography of purified Rv1738 (red), size standards (44 and 17 kDa) are in black. **(B)** Cell-free ribosome activity with increasing concentrations of Rv1738 incubated with 240 μM of **1**, **5**, **C5**, or **9c**. Luminescence (RLU) from the activity of the luciferase protein translated from the nano-luciferase mRNA, served as a surrogate measure of ribosomal activity.

We focused first on shortlisted proteins (Table 2) that are unannotated: Rv0250, Rv1738, and Rv0786. Of these, only Rv1738 has a published crystal structure (Bunker et al., 2015). The dimer of Rv1738 bears a striking similarity to the structure of *E. coli’s* hibernation promoting factor, which plays a role in imposing an NR state. Accordingly, we purified recombinant Rv1738 protein (Supplementary Figure S1), which eluted in dimeric form (Figure 5A). When incubated with purified Mtb ribosomes, Rv1738 inhibited up to 80% of translation of luciferase mRNA in a concentration-dependent manner. Thus, Rv1738 appears to be an Mtb ortholog of hibernation factor. However, the addition of **1**, **5**, or **C5** did not alter this interaction (Figure 5B).

Rv0786 is an uncharacterized conserved protein. Although the percent sequence identity was below 25%, 66% of Rv0786’s structure was predicted with 100% confidence by Phyre2 3D software (Kelley et al., 2015) to share similarities with class B3 metallo-β-lactamases (Supplementary Figure S2A). Rv0786 contained the metal binding site common to Class 3 β-lactamases such as FEZ-1 and AIM-1 (Supplementary Figure S2B) (Sun et al., 2018). To test the role of Rv0786 in the survival of the bacteria under NR conditions, a transposon mutant of *rv0786* from a predicted loss-of-function library was prepared in the Hung laboratory at the Broad Institute. The *rv0786* mutant showed neither decreased survival nor a change in response to **5** compared to the WT strain (Supplementary Figures S2C,D).

**FIGURE 6:**
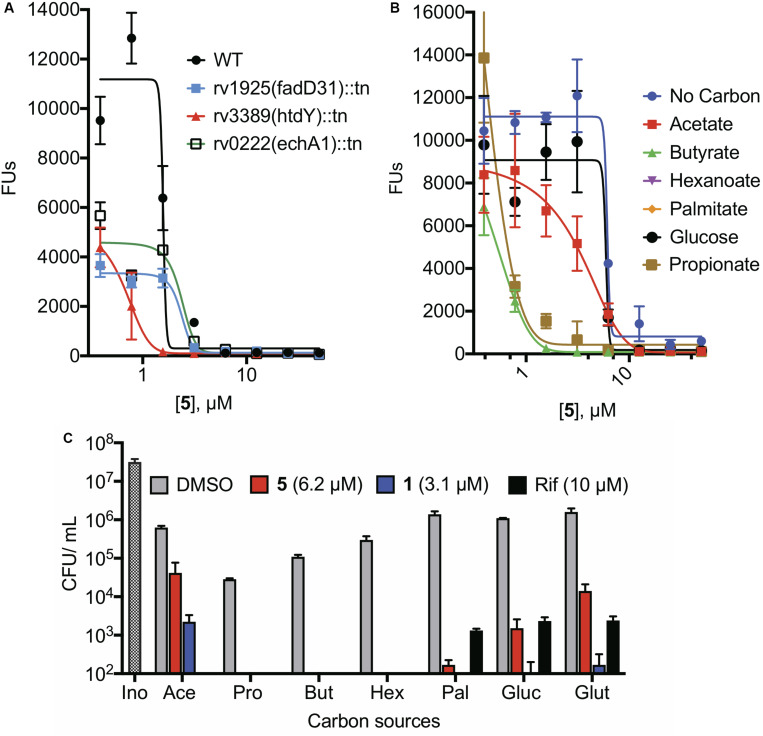
Effects of fatty acid carbon sources on the activity of NR-active cephalosporins under NR conditions. **(A)** NR activity of 5 against transposon mutants in genes encoding enzymes involved in fatty acid metabolism. **(B)** NR activity of 5 using 0.05% acetate, 0.05% propionate, 0.05% butyrate, 0.015% hexanoate, 0.00025% palmitate, 0.2% glucose, or 0.2% glutamate as the sole carbon source in NR media. **(C)** When exposed to a concentration of compound equivalent to its CARA_MBC_, reduction in viability after 7 days of treatment using various carbon sources in the NR media.

Loss-of-function transposon mutants from the Hung library for the following binding partners showed no change in the survival of the bacteria to the NR assay conditions. The mutants that we tested included those disrupted in the genes encoding the universal stress proteins Rv1996, Rv2005c, and Rv2326c (Supplementary Figures S2C,D, and data not shown), known or potential fatty acid metabolism enzymes Rv3224 (possible short chain dehydrogenase), Rv0222 (EchA1), and Rv3389 (putative HtdY) (Figure 6A and Supplementary Figures S3A,B), LldD (possible L-lactate dehydrogenase), and PdxH, pyridoxine 5′-oxidase (Supplementary Figures S3A,B).

The 4-stress NR model provides butyrate as the carbon source. Because there is a high degree of redundancy among enzymes involved in β-oxidation (Williams et al., 2011), we explored the pathway as a whole by altering the carbon source in the NR model. The carbon source markedly affected the susceptibility of Mtb to the NR-active cephalosporins (Figures 6B,C). Compared to standard NR conditions, in which the carbon source was butyrate, NR conditions lacking any carbon source reduced the susceptibility of Mtb both to the NR-active cephalosporins and to rifampicin (Supplementary Figure S4B). Compared to the control in which no carbon source was added, butyrate, propionate, and, to a lesser extent, hexanoate increased Mtb’s susceptibility to **5**. In contrast, acetate increased Mtb’s resistance to **5** and **1** without altering susceptibility to rifampicin (Figure 6C) compared to the no-carbon control. The non-fatty acid carbon sources glutamate and glucose reduced the cidality of both **5** and rifampicin. These complex responses did not allow us to conclusively identify fatty acid oxidation as a pathway target.

Finally, we generated a knockout of *htpG*, which encodes a predicted protein chaperone (Supplementary Figures S5A,B) and obtained the knockout strain of *fecB*, a putative iron(III) dicitrate-binding protein (Xu et al., 2017). The strain deficient in FecB was slower than WT to recover after 3 days under NR conditions (Supplementary Figure S5C). Also striking, the Δ*htpG* strain had a fourfold decrease in CARA_MBC_ when exposed to **1** and **5** (Supplementary Figure S5D) compared to the wild type. We purified recombinant HtpG and used differential scanning fluorimetry to determine if **5** binds HtpG. In contrast to HtpG’s natural ligand, ADP, **5** did not alter the melting temperature of HtpG (Supplementary Figure S5E). This may indicate that conditions leading to binding in the intact cell were not recapitulated *in vitro* or that, indeed, there is no ligand-protein interaction.

## Discussion

The NR-active cephalosporins studied here were inactive on isolated Ldts and pulled down no Ldts or PBPs by ABPP. In contrast, ABPP identified at least 30 other cephalosporin binding proteins reproducibly and selectively. While one putative protein partner is predicted to be a metallo-β-lactamase, no other β-lactam-associated proteins were among the 30 candidate targets. Enzymes of fatty acid metabolism, universal stress proteins, and ribosome-associated proteins stood out as clusters, and several other enzymatic pathways were represented.

The isolation of seemingly unrelated chemical classes is not uncommon in the ABPP approach. A chemical proteomic strategy was used to isolate the targets of 10 alkyne β-lactone analogs in the proteomes of several Gram-negative and Gram-positive bacteria (Bottcher and Sieber, 2008), leading to isolation of acetyl-CoA hydrolases, several β-ketoacyl carrier protein synthases, ligases, and oxidoreductases, among other enzymes. Another study used several alkyne β-lactam probes including two penems containing an alkyne moiety either at the amino group (AmpN) or the carboxyl group (AmpC) and one cephalosporin with the alkyne at the amino group (CephN) (Staub and Sieber, 2008). Using *Pseudomonas putida* and two Gram-positive soil bacteria (*Listeria welshimeri* and *Bacillus licheniformis*) for their ABPP experiments, Staub and Sieber obtained similar results to ours in that AmpC, unlike AmpN, more readily bound non-PBPs. Even though the CephN contained a free carboxylic acid, in *B. licheniformis*, it bound a non-PBP protein called DltD, a membrane-bound thioesterase involved in lipoteichoic acid synthesis (Debabov et al., 2000; Staub and Sieber, 2008). Along with the high-molecular-weight and low-molecular-weight members of the PBP superfamily, Staub and Sieber also isolated β-ketoacyl-acyl carrier protein synthase III from multiple species with alkyne β-lactam probes. Thus, ABPP with β-lactam probes can identify non-canonical targets.

Several potential protein binders of **1** and **5** have roles in the Mtb stress response. Mutations in *fecB* rendered Mtb hypersusceptible to diverse classes of antibiotics, including vancomycin, isoniazid, ethambutol, and meropenem (Xu et al., 2017). Despite its annotation as an iron (III) dicitrate-binding periplasmic lipoprotein, knockout of *fecB* did not alter the mutant’s ability to grow in iron-limiting or iron-replete conditions; however, the Δ*fecB* mutant was more permeable to ethidium bromide and fluorescently tagged vancomycin (Xu et al., 2017). The *fecB* mutant’s weakened cell wall barrier could explain the increase in susceptibility to a wide range of antibiotics and perhaps also to nitroxidative stress. HtpG, the bacterial homolog of the eukaryotic chaperone Hsp90, has an important role in the adaptation of *E. coli* to environmental stresses, including incubation at high temperature (Grudniak et al., 2013) and host cell infection (Garcie et al., 2016); however, in Mtb, much remains to be learned about the nature and function of this chaperone (Lupoli et al., 2018). Using DSF, we did not detect interaction of compounds **1** and **5** with HtpG. In intact cells, the disruption in proteostasis caused by the loss of *htpG*, along with additional stresses after addition of β-lactam and nitric oxide, might lead to the increased sensitivity seen in the *htpG* knockout versus wild-type Mtb in the presence of compound.

*Rv1738*, encoding a 94-amino-acid protein considered non-essential for growth (DeJesus et al., 2017), is the most abundantly upregulated gene under hypoxic conditions (Sherman et al., 2001). *Rv1738* is also upregulated during NO stress (Voskuil et al., 2003). The crystal structure indicates that the dimer bears a close structural similarity to theβαβββα motif of hibernation promoting factor (Bunker et al., 2015). Purified Rv1738 appeared dimeric when eluted from a size exclusion column. When bacteria approach a dormant state, protein synthesis is retarded by the inactivation of their ribosomes. Ribosome modulation factor and hibernation factor promote and stabilize dimerization of the ribosomal subunits. The binding of these small proteins to the head domain of the 30S ribosomal subunit impedes the interaction of tRNAs and mRNAs with the 16S ribosomal RNA and induces a conformational change of the 30S subunit (Polikanov et al., 2012). Cryo-EM structures of ribosomes isolated from stationary phase *Mycobacterium smegmatis* showed 100S dimers but did show 70S ribosomes bound to a hibernation factor at the t-RNA binding site (Mishra et al., 2018). Our findings indicate that Rv1738 can behave like a hibernation promoting factor *in vitro* but how it does so remains to be determined. We did not find evidence that NR-active cephalosporins inhibit the action of Rv1738 *in vitro*, but we cannot exclude that they might do so in the intact cell.

A meta-analysis of the *M. tuberculosis* transcriptomic landscape revealed that several genes encoding candidate binding proteins, including *rv2005c*, *rv199c*, and *rv2623* are upregulated when the bacilli are under stress conditions. *Rv2005c* encodes a universal stress protein and is upregulated under hypoxic conditions (Parvati Sai Arun et al., 2018). Rv2005c and Rv1996c belong to the same family of proteins as Rv2623, another universal stress protein. *Rv2623* is upregulated during NO exposure or hypoxic stress (Glass et al., 2017). Rv2623 has a role in the establishment of chronic infection in mice and overexpression slowed the growth of the cells (Drumm et al., 2009). However, the transposon mutants of *rv2623*, *rv1996*, or *rv2005* had no discernable phenotype in our NR 4-stress model. This does not exclude their possible collective relevance, given that the biological roles of Rv2005c and Rv1996c appear to be functionally redundant (Hingley-Wilson et al., 2010).

There is likely redundancy among the binding partners involved in fatty acid metabolism. Exposure to NO reportedly increased the expression of AcpM and other enzymes involved in fatty acid biosynthesis, and β-oxidation enzymes were induced by host intracellular environments (Parvati Sai Arun et al., 2018). This is consistent with the importance of host-derived fatty acids as carbon sources for Mtb during infection (McKinney et al., 2000; Schnappinger et al., 2003). Several studies have highlighted a link between central-carbon metabolism and antibiotic susceptibility and tolerance (Hicks et al., 2018; Lee et al., 2019) in which the bacterium altered its carbon flux to increase its survival during antibiotic stress. Because carbon metabolism in Mtb is distinctive and the metabolic fate of different carbon sources can differ (de Carvalho et al., 2010), further studies are needed to understand how these non-traditional cephalosporins affect the compartmentalization of these carbon sources, particularly fatty acids. At an individual level, the functional loss of *fadD31* and *echA1* did not appear to affect the efficacy of **5** or **1** (data not shown), but there appears to be redundancy among genes in these classes (Williams et al., 2011); indeed, Mtb has over 100 genes annotated to be involved in the five reactions that convert fatty acids into acetyl-CoA.

In sum, the isolation of several protein families that are important in Mtb stress responses or adaptation to the host environment and that have mutually redundant members suggests that cephalosporins **1** and **5** may kill NR Mtb through collective inhibition of more than one target. However, it remains possible that there is a single, functionally relevant target we did not identify by ABPP due its low abundance or tight association with insoluble components, and it remains possible that the high abundance of several of the protein binders could explain their presence as pulldown clients. Moreover, one or more of the binding proteins that are essential under replicating conditions could represent the functionally relevant target or targets under NR conditions if the NR-active cephalosporins inhibit such target(s) partially and NR conditions disable the same target(s) as well, the combined effect being necessary to kill Mtb.

## Materials and Methods

### Materials

Compounds **1**, **5**, and **9c** were synthesized as described below. The biotin-diol-azide linker was obtained from Click Chemistry Tools (Scottsdale, United States). Activated charcoal, sodium resazurin, sodium periodate, Triton X-114, isoniazid, rifampicin (Rif), and dimethyl sulfoxide (DMSO) were from Sigma-Aldrich, United States. The tris-(2-carboxyethyl)phosphine HCl (TCEP) and streptavidin-agarose beads used for enrichment and purification were from Thermo Fisher Scientific, United States.

### Synthesis of Alkyne Probes

All starting β-lactams were prepared as described (Gold et al., 2016b).

#### *n*-Prop-2-yn-1-yl (6*R*,7*R*)-7-(2-(4-methoxyphenoxy) acetamido)-3-methyl-8-oxo-5-thia-1-azabicyclo [4.2.0]oct-2-ene-2-carboxylate (C1a)

To a solution of (6*R*,7*R*)-7-(2-(4-methoxyphenoxy)acetamido)-3-methyl-8-oxo-5-thia-1-azabicyclo[4.2.0]oct-2-ene-2-carboxylic acid (2.33 g, 6.17 mmol) in methyl isobutyl ketone (195 ml), a solution of potassium 2-ethylhexanoate hydrate (1.69 g, 9.25 mmol) in 1-butanol (37.5 ml) was added. The reaction became clouded. Hexanes were added until an off-white solid crashed out of solution. The precipitate (2.57 g, 6.17 mmol) was collected via filtration and used without further purification. To a suspension of this precipitate (2.57 g, 6.17 mmol) in DMF (30.9 ml, 0.20 M), 3-iodoprop-1-yne (4.79 ml, 30.9 mmol) was added. The mixture was allowed to stir at RT for 16 h. Solvent was removed and the residue was purified via silica gel MPLC to give **C1a** (1.78 g, 69%) as a white solid. Purity: 92%. ^1^H NMR (400 MHz, DMSO-*d*_6_) δ 9.00 (d, *J* = 8.3 Hz, 1H), 6.90–6.81 (m, 4H), 5.67 (dd, *J* = 8.3, 4.7 Hz, 1H), 5.12 (d, *J* = 4.7 Hz, 1H), 4.94–4.77 (m, 2H), 4.60–4.49 (m, 2H), 3.69 (s, 3H), 3.63–3.55 (m, 2H), 3.32 (s, 1H), 2.05 (s, 3H). ^13^C NMR (101 MHz, DMSO-d_6_) δ 168.7, 164.2, 161.3, 153.8, 151.8, 133.4, 121.3, 115.5, 114.5, 78.1, 77.9, 66.9, 58.8, 57.3, 55.4, 52.6, 29.2, 19.4. HR-MS (ESI): calculated for C_20_H_21_N_2_O_6_S [M + H]^+^ 417.1115; found, 417.1088.

#### Pent-4-ynoyl chloride

To a solution of pent-4-ynoic acid (1.55 g, 15 mmol) in DCM (15 ml) was added three drops of DMF at 0°C, followed by slow addition of oxalyl chloride (3.86 ml, 45 mmol). The reaction mixture was stirred at RT for 2 h, and then the solvent was removed under reduced pressure to afford the product (1.66 g, 95%) as an oil and used without purification.

#### (6*R*,7*R*)-3-Methyl-8-oxo-7-(pent-4-ynamido)-5-thia-1-azabicyclo[4.2.0]oct-2-ene-2-carboxylic acid

To a suspension of 7-ADCA (3.21 g, 15.0 mmol) in water (100 ml), NaHCO_3_ (1.26 g, 15.0 mmol) and acetone (12.0 ml) were added, followed by the addition of pent-4-ynoyl chloride (1.75 g, 15.0 mmol). The reaction was stirred at RT for 16 h. DCM (100 ml) was added and the reaction mixture was acidified using 6N HCl to pH 2. The organic layer was removed and the aqueous portion was extracted again with DCM (100 ml). The organic extracts were combined, washed with brine, and dried over MgSO_4_. Solvent was removed after filtration and the solid was suspended in ether and stirred for 8 h. The product was isolated through vacuum filtration as an off-white solid (3.43 g, 78%) and used without further purification.

#### *n*-Propyl (6*R*,7*R*)-3-methyl-8-oxo-7-(pent-4-ynamido) -5-thia-1-azabicyclo[4.2.0]oct-2-ene-2-carboxylate (N1)

0.13 g, 64% yield. Purity: 92%. A similar procedure to C1a was used for the synthesis with corresponding acid and 1-iodopopane. ^1^H NMR (400 MHz, CDCl_3_) δ 6.74 (d, *J* = 8.8 Hz, 1H), 5.79 (dd, *J* = 8.8, 4.7 Hz, 1H), 4.96 (d, *J* = 4.7 Hz, 1H), 4.27–4.09 (m, 2H), 3.49 (d, *J* = 18.3 Hz, 1H), 3.21 (d, *J* = 18.3 Hz, 1H), 2.57–2.46 (m, 4H), 2.13 (d, *J* = 0.9 Hz, 3H), 2.04–2.00 (m, 1H), 1.75–1.66 (m, 2H), 0.96 (t, *J* = 7.4 Hz, 3H). ^13^C NMR (101 MHz, CDCl_3_) δ 171.4, 164.5, 162.2, 131.2, 122.7, 82.5, 69.6, 67.3, 58.9, 57.1, 34.8, 30.2, 21.8, 20.0, 14.6, 10.4. HR-MS (ESI): calculated for C_16_H_21_N_2_O_4_S [M + H]^+^ 337.1217; found, 337.1228.

#### *N*-Hydroxypent-4-ynimidamide

To a solution of hydroxylamine hydrochloride (1.05 g, 15.0 mmol) in water (5 ml), sodium hydroxide (0.60 g, 15.0 mmol) was added. The resulting solution was added to pent-4-ynenitrile (1.19 g, 15.0 mmol) in about 2 min. The mixture was stirred at RT for 2 days. Solvent was removed and residue was treated with EtOH and the resulting suspension was filtered. The filtrate was concentrated *in vacuo* to afford product (0.84 g, 50%) as a white solid, which was used without further purification.

#### *N*-((6*R*,7*R*)-2-(3-But-3-yn-1-yl)-1,2,4-Oxadiazol-5-yl)-3-methyl-8-oxo-5-thia-1-azabicyclo[4.2.0]oct-2-en-7-yl)-2-phenylacetamide (C5)

To a solution of 2,4-dinitrophenol (0.92 g, 5.00 mmol) in DCM (20 ml), (6*R*,7*R*)-3-methyl-8-oxo-7-(2-phenylacetamido)-5-thia-1-azabicyclo[4.2.0]oct-2-ene-2-carboxylic acid in a minimal amount of 1,4-dioxane was added, followed by the addition of 1,3-diphenylcarbodiimide (0.97 g, 5.00 mmol) in DCM (10 ml). The mixture was stirred at RT for 30 min. After the filtration, *N*-hydroxypent-4-ynimidamide was added to the filtrate and the mixture was stirred at RT overnight. The mixture was then washed twice with sat. aq. NaHCO_3_, filtered, and concentrated in vacuum to afford the residue, which was purified via silica gel MPLC (100% EtOAc) to give the intermediate (0.18 g) as a white solid, which was heated at 120°C in a vacuum oven overnight. Purification via silica gel MPLC afforded the desired product (68 mg, 39%, two steps) as a white solid. Purity: 98%. ^1^H NMR (400 MHz, DMSO-*d*_6_) δ 9.12 (d, *J* = 8.2 Hz, 1H), 7.33–7.19 (m, 5H), 5.72 (dd, *J* = 8.2, 4.7 Hz, 1H), 5.20 (d, *J* = 4.7 Hz, 1H), 3.75–3.47 (m, 4H), 2.97 (t, *J* = 7.1 Hz, 2H), 2.82 (t, *J* = 2.7 Hz, 1H), 2.63 (td, *J* = 7.2, 2.7 Hz, 2H), 2.15 (s, 3H). ^13^C NMR (101 MHz, DMSO-*d*_6_) δ 171.0, 170.2, 169.1, 165.2, 135.8, 132.7, 129.0, 128.2, 126.5, 115.7, 82.5, 72.2, 59.2, 57.6, 41.6, 29.0, 24.9, 19.8, 15.7. HR-MS (ESI): calculated for C_21_H_21_N_4_O_3_S [M + H]^+^ 409.1329; found, 409.1364.

#### *N*-((6*R*,7*R*)-3-Methyl-2-(3-Methyl-1,2,4-oxadiazol-5-yl)-8-oxo-5-thia-1-azabicyclo[4.2.0]oct-2-en-7-yl) pent-4-ynamide (N5)

A similar procedure to that described for **C5** was used starting with (6*R*,7*R*)-3-methyl-8-oxo-7-(pent-4-ynamido)-5-thia-1-azabicyclo[4.2.0]oct-2-ene-2-carboxylic acid and acetonitrile. Obtained 0.137 g (15% yield for the last two steps). Purity: 100%. ^1^H NMR (400 MHz, CDCl_3_) δ 6.62 (d, *J* = 8.6 Hz, 1H), 5.86 (dd, *J* = 8.6, 4.7 Hz, 1H), 5.08 (d, *J* = 4.7 Hz, 1H), 3.58 (d, *J* = 18.4 Hz, 1H), 3.33 (d, *J* = 18.4 Hz, 1H), 2.58–2.46 (m, 4H), 2.45 (s, 3H), 2.22 (s, 3H), 2.02 (t, *J* = 1.7 Hz, 1H). ^13^C NMR (101 MHz, DMSO-*d*_6_) δ 171.4, 170.2, 167.3, 165.3, 132.4, 115.5, 83.5, 71.5, 59.1, 57.5, 33.7, 29.0, 19.8, 14.1, 11.4. HR-MS (ESI): calculated for C_15_H_17_N_4_O_3_S [M + H]^+^ 333.1021; found, 333.0981.

### Bacterial Strains and Growth Conditions

Actively growing WT *Mycobacterium tuberculosis* strain H37Rv (*Mtb*, ATCC 25618) was grown in Middlebrook 7H9 broth supplemented with 10% oleic acid, dextrose and catalase (OADC) (BD Difco, United States), 0.5% glycerol, and 0.02% Tyloxapol (Sigma) and incubated at 5% CO_2_ and 20% O_2_ at 37°C. H37Rv transposon mutants of Mtb *rv1925*, *lldD*, *rv3389*, *rv1996*, *rv2005*, *rv0786*, *rv3224*, *pdxH*, *fadD31*, *rv2623*, and *rv0222* H37Rv [a generous gift from Deborah Hung (Barczak et al., 2017)] were grown in Middlebrook 7H9 containing 25 μg/ml kanamycin. Non-replicating assays were done using a reported 4-stress model (Warrier et al., 2015; Gold et al., 2016b). In brief, replicating cells were washed twice and diluted to an OD_580_ of 0.1 in non-replicating (NR) medium: modified Sauton’s base (0.05% KH_2_PO_4_, 0.05% MgSO_4_, and 0.005% ferric ammonium citrate at pH 5.0) with 0.02% tyloxapol, 0.5% bovine serum albumin (BSA), 0.0001% ZnSO_4_, and 0.05% (NH_4_)_2_SO_4_ 0.085% NaCl; butyrate (0.05%) was used as the carbon source and a flux of nitric oxide was generated by adding 0.5 mM NaNO_2_ to the acidified medium. For conditions involving different carbon sources, the butyrate was replaced with 0.05% acetate, 0.05% propionate, 0.015% hexanoate, 0.00025% palmitate, 0.2% glucose, or 0.2% glutamate, and the pH was adjusted to 5.0. The cells were incubated with 5% CO_2_ and 1.0% O_2_ at 37°C. Unless otherwise stated, cells were exposed to compounds for 7 days for the following assays: minimum inhibitory concentration (MIC), charcoal agar resazurin assay (CARA), or colony-forming unit (CFU).

### Compound Activity (CFU/ml, MIC, and CARA)

Bacilli were enumerated by plating serially diluted bacterial cultures on 7H11 agar (BD Difco, Franklin Lakes, NJ, United States) plates supplemented with 0.5% glycerol and 10% OADC. MICs were determined by serially diluting compounds in DMSO and dispensing into 96-well plate with 200 μl of cells. The final DMSO concentrations did not exceed 1%. Compound dilutions were prepared the same day of experiments. For the replicating MIC assays, bacterial suspensions were diluted to an OD_580_ of 0.01 and added to the 96-well plate containing the compound. The MIC of a compound was the lowest concentration leading to ≥90% inhibition of the cell growth compared to cells treated with vehicle (DMSO). Growth was determined by OD_580_. For non-replicating cells, the NR MIC was determined as described (Warrier et al., 2015) by resuspending cells from the NR plates and using 1/20 of the volume to inoculate a second plate containing replication-supporting, supplemented 7H9 broth. The latter plates were incubated under replicating conditions for 10–12 days before recording the OD_580_. CARA plates were prepared and assayed as described (Gold et al., 2015a, 2016a). In brief, after 7 days of compound exposure, cells in the assay plate were resuspended and 10 μl were used to inoculate CARA plates containing agar with 4 g/L activated charcoal. After incubating the CARA plates for 7 days (replicating assay) or 12–14 days (NR assay), growth was estimated by adding 50 μl of 0.01% resazurin in PBS with 0.02% Tyl to each well of the CARA plates. The CARA_MBC_ was defined as the lowest concentration of compound that reduced fluorescence to ≤1% of that in DMSO control wells for each strain, which corresponds to a difference in viable cell number of ≥2–3 log_10_ CFU (Gold et al., 2016a).

### Ldts and BlaC Kinetics

Production, purification, and activity of Mtb Ldt_Mt__1_ and Ldt_Mt__2_ and *E. faecium* Ldt_fm_ were carried out as previously described (Cordillot et al., 2013; Edoo et al., 2017). Briefly, BlaC hydrolysis of **1**, **5**, and **9c** was determined by incubating 100 μM of each compound with 50 and 500 nM of the enzyme in 100 mM MES buffer at pH 6.4. Inhibition of BlaC-mediated hydrolysis of nitrocefin was tested by incubating 2 nM of BlaC in the presence of increasing concentration of **1**, **5**, and **9c** and 50 μM of nitrocefin. To account for time-dependent inhibition, 1 nM of BlaC was incubated for 2 h with the compounds prior to the addition of nitrocefin. In the case of Ldt_Mt__1_ and Ldt_Mt__2_, all kinetic tests were conducted in 100 mM sodium phosphate at pH 6.0 at 20°C. To asses Ldt hydrolysis of the compounds, 50 μM of **1**, **5**, or **9c** was incubated with increasing concentration of Ldt (0, 1, 5, and 10 μM). Inhibition of enzyme activity was determined by incubating 5 μM of the Ldt enzymes in the presence of test compounds (0, 25, 50, and 100 μM) and 50 μM of nitrocefin. Assays were performed using enzymes incubated for 30 min with the compounds prior to the addition of nitrocefin. Stopped flow kinetics were conducted using Ldt_fm_ in 100 mM sodium phosphate at pH 6.0 at 10°C. Ldt_fm_ (15 μM) and 20 μM of 1, 5, or imipenem were mixed, and the acylated form of Ldt_fm_ was monitored by mass spectrometry.

### Bacterial Proteome Ligand Binding

Mtb cells were washed with PBS containing 0.02% Tyloxapol, diluted to an OD_580_ of 0.8–1.0 in 30-ml cultures, and pre-adapted to non-replicating conditions for 24 h. Following the pre-adaptation to NR conditions, 50–70 μM of compounds or vehicle control (DMSO) was added to the cultures and placed back in the 37°C incubator. After a 24-h exposure to compounds, cells were washed 2× with PBS containing 0.02% Tyloxapol, harvested, and stored at −80°C until ready for lysis. The protocol as shown in Figure 3 was conducted twice.

Extraction buffers consisted of PBS containing Roche Protease Inhibitor Cocktail and 2% Triton X-114 in PBS (kept at 4°C) as previously described (Malen et al., 2010) with modifications. Samples were resuspended in ∼720 μl of PBS and transferred to bead beating tubes containing 500 μl of zirconium beads. Samples were maintained ice cold. Bacteria were lysed by bead beating and centrifuged for 5 min at 1400 × *g* to remove beads and unlysed cells. There was approximately 800 μl of lysate. Eighty microliters of PBS-20% Triton X-114 solution was added to each sample. Samples rotated gently at 4°C overnight. To remove insoluble material, samples were centrifuged at 4°C at 8600 × *g* for 10 min. In contrast to published protocols, samples were maintained chilled such that the detergent (membrane) fraction and aqueous phase were maintained in solution. In our experience, phase separation reduced the final yield of proteins.

### Probe Conjugation, Purification and Enrichment of Target Proteins

Based on published general click chemistry protocols (Speers and Cravatt, 2009; Yang et al., 2013), samples were diluted with PBS to halve the detergent concentration (<1%) prior to incubating them overnight at 4°C with streptavidin beads. Once biotinylated proteins were removed, protein concentrations were determined using the Pierce BCA Protein Assay Kit (Thermo Fisher Scientific, United States) and samples were diluted to the same protein concentration (∼1 mg/ml). The samples were divided into 500-μl aliquots. For Cu(I) catalyzed Huisgen’s azide alkyne cycloaddition reactions, the following was added to each sample: 5.0 μl of 5 mM diol-biotin-azide (50 μM final), 11.3 μl 50 mM TCEP (1 mM final), 7.0 μl 1.7 mM TBTA (tris[(1-benzyl-1H-1,2,3-triazol-4-yl)methyl]amine) (50 μM final), and 11.3 μl of 50 mM CuSO_4_ (1 mM final). Samples were vortexed between each step and kept in the dark. The reaction proceeded for 1 h at RT with vortexing after 30 min of incubation. Some proteins precipitated out of solution. Accordingly, samples were centrifuged for 15 min at 10,000 × *g* and the pellet was set aside as the “precipitate.” The pellet was washed with 500 μl of cold methanol and resuspended in 2.5% SDS to solubilize the proteins. The precipitate fractions were diluted to <0.2% SDS prior to the addition of the streptavidin beads. To stop the reaction in the supernatant or “soluble fraction” and remove excess reagents, samples were run through a PD-10 column (Bio-Rad). One hundred microliters of streptavidin-agarose bead slurry was added to each sample and incubated overnight at 4°C. The beads were washed 2× with 1 volume of PBS containing 0.1% Triton X-100 including one O/N wash. This was followed by three washes with PBS. Proteins were eluted from the beads by resuspending the slurry in 10 mM sodium periodate in 100 mM sodium phosphate buffer, pH 7.4. The samples were incubated for 30 min in the dark with rotation. Glucose (20 mM final) was added to quench the reactions. Samples were desalted using Zeba spin columns (Thermo Fisher Scientific). Samples were concentrated by TCA precipitation and analyzed by silver staining, Western blot, and mass spectrometry.

### Rv1738 Purification and Ribosomal Inhibition

Rv1738 and ribosome functional assays were performed as previously described (Li et al., 2015) in which Rv1738 was cloned into p1602-dest (Life Technologies) with a C-terminal His6x tag on Rv1738. Rv1738 was purified with Ni^2+^ chromatography column (GE Healthcare), followed by size exclusion chromatography. A superdex 75 column (GE Healthcare) was used for size exclusion chromatography in a buffer containing 20 mM Tris–Cl (pH 7.5) and 150 mM NaCl, and the protein profile was compared with protein molecular size standards.

In order to purify *Mtb* ribosome, *Mtb* strain MC^2^7000 was grown in 7H9 medium supplemented with 10% OADC supplement (BD), 0.5% glycerol, 0.05% Tween-80, and 50 μg/ml pantothenic acid at 37°C until an OD_600_ of 1.0. Harvested cells were lysed in a bead beater (BioSpec) in lysis buffer [20 mM Tris–HCl (pH 7.5), 100 mM NH_4_Cl, 10 mM MgCl_2_, 0.5 mM EDTA, and 6 mM 2-mercaptoethanol]. The cell lysate was clarified by centrifugation at 30,000 × *g* for 1 h. The supernatant was pelleted in a sucrose cushion buffer [20 mM HEPES (pH 7.5), 1.1 M sucrose, 10 mM MgCl_2_, 0.5 M KCl, and 0.5 mM EDTA] at 40,000 rpm in a Beckman Type 45Ti rotor for 20 h. The pellet was resuspended in a buffer of 20 mM Tris–HCl (pH 7.5), 1.5 M (NH_4_)_2_SO_4_, 0.4 M KCl, and 10 mM MgCl_2_. The suspension was then applied to a hydrophobic interaction column (Toyopearl Butyl-650S) and eluted with a reverse ionic strength gradient from 1.5 to 0 M (NH_4_)_2_SO_4_ in a buffer containing 20 mM Tris–HCl (pH 7.5), 0.4 M KCl, and 10 mM MgCl_2_. The eluted ribosome peak was changed to re-association buffer [5 mM HEPES-NaOH (pH 7.5), 10 mM NH_4_Cl, 50 mM KCl, 10 mM MgCl_2_, and 6 mM 2-mercaptoethanol] and concentrated before loading on top of a 10–40% linear sucrose gradient and centrifuged in a Beckman SW28 rotor at 19,000 rpm for 19 h. The 70S fractions were concentrated to about *A*_260_ = 300 after removal of the sucrose. *Mtb* S30 cell-free extract was prepared according to methods (Swartz et al., 2004) and S100 extract was prepared by removing endogenous ribosomes from the S30 extract. The 15 μl of the S100 extract that includes translation factors such as initiation, elongation, termination, recycling factors, and aminoacyl tRNA synthetase was mixed with 5 μl 10 × salt buffer (2 M potassium glutamate, 0.8 M ammonium acetate, and 0.16 M magnesium acetate), 1 mM each of the 20 amino acids, 33 mM PEP, and 2% poly(ethylene glycol) 8000. Rv1738 or the compounds or their mixtures with ribosomes were added to the reactions prior to the master mix and mRNA. The reaction was started by the addition of nano-luciferase mRNA (200 ng in 2 μl) and 5 μl of 5× master mix (286 mM HEPES-KOH, pH 7.5, 6 mM ATP, 4.3 mM GTP, 333 μM folinic acid, and 853 μg/ml tRNA) to reach a final volume of 50 μl. The reaction was allowed to proceed for 40 min at 37°C and the luminescent signal was detected by the addition of 20 μl of the nano-luciferase substrate furimazine. **1**, **5**, **9c**, and **C5** were first tested in the cell-free translation assay to ensure that the compounds alone did not inhibit ribosomal activity. To test the impact that the compounds had on Rv1738-mediated inhibition of translation, we tested several combinations: ribosomes and 10 μM Rv1738 were incubated for 10 min followed by the addition of 240 μM of the compounds; ribosomes and 240 μM compounds were incubated for 10 min followed by the addition of 10 μM of Rv1738; 10 μM of Rv1738 was incubated with 240 μM compounds before mixing with ribosomes; and finally, the reaction was carried out in which 240 μM compounds and ribosome were co-incubated first, followed by the titration of Rv1738.

### *HtpG* Characterization and Differential Scanning Fluorimetry (DSF)

*Mycobacterium tuberculosis*Δ*htpG* was generated using a suicide plasmid approach, which enlists Gateway cloning techniques and vectors. Briefly, ∼1100 bp fragments, including regions upstream and downstream of *htpG* (*rv2299c*), were amplified from chromosomal *M. tuberculosis* H37RvN DNA using primers 1–4 (Supplementary Table S3). The fragments were cloned into pDE43-XSTS (a temperature-sensitive plasmid) containing a zeocin resistance cassette, which was amplified from pGMCZ-PrpoB using primers 9 and 10 to produce pKO-XSTS-htpG-tb (Pelicic et al., 1997; Kim et al., 2011; Ganapathy et al., 2015). *M. tuberculosis* H37RvN was transformed with pKO-XSTS-htpG-tb and plated on 7H10 agar containing zeocin (50 μg/ml), followed by incubation at the permissive temperature of 37°C. Resulting transformants were then inoculated into 7H9 complete (with Tween-80) containing zeocin (25 μg/ml) at 37°C and grown to stationary phase. Cells were periodically plated on 7H10 agar with zeocin (50 μg/ml) containing 10% sucrose and incubated at the restrictive temperature of 40°C. Pyrocatechol (0.5 M) was added to plates that contained colonies and white colonies were inoculated into 7H9 complete (with Tween-80) containing zeocin (25 μg/ml) and grown at 37°C prior to purification of DNA and confirmation of allelic exchange by Southern blot. Southern blot of selected clones was performed using a DNA probe (∼400 bp) generated with primers 5–6 (Supplementary Table S3) in order to analyze genomic DNA from *M. tuberculosis* wild type and candidate Δ*htpG* clones digested with *Pvu*II (Supplementary Figure S5). Deletion of *htpG* and insertion of zeocin resistant cassette were confirmed by whole genome sequencing. For overexpression of HtpG in Mtb Δ*htpG*, we generated pMCH_pH60_SD_htpG using primers 11 and 12 (Supplementary Table S3), an episomal vector that expresses *htpG* under the control of the *hsp60* promoter, as has been previously described (Kim et al., 2011; White et al., 2018).

Recombinant *M. tuberculosis* HtpG was cloned and purified using previously described methods (Lupoli et al., 2016). The overexpression plasmid was constructed by overlap extension PCR cloning techniques (Bryksin and Matsumura, 2010) with pET-His-SUMO plasmid (Addgene #29711) and primer pair 7–8 (Supplementary Table S3). DNA samples were purified using a PCR purification kit (Qiagen) and transformed into Mach1 competent cells (Invitrogen). After confirmation of gene insertion by DNA sequencing, the selected plasmid was transformed into Rosetta2 competent cells (Novagen) for overexpression and purification using similar techniques to those previously described for other mycobacterial chaperones (Lupoli et al., 2016). For expression, *E. coli* Rosetta2 cultures containing His_6_-SUMO-HtpG overexpression plasmid were grown in LB medium supplemented with 50 μg/ml carbenicillin, 30 μg/ml chloramphenicol, and 0.1% glucose, and then used to inoculate 0.5 L of LB medium (1:100) supplemented with 50 μg/ml carbenicillin and 30 μg/ml chloramphenicol at 37°C and grown to OD_600_ = 0.3–0.4 with shaking. Cells were cooled to 16°C and grown to OD_600_ ∼ 0.5 before induction with 0.01 mM isopropyl-β-D-thiogalactoside (IPTG) for 18 h with shaking. Cells were harvested by centrifugation (3100 × *g*, 10 min, 4°C) and pellet was resuspended on ice with 15 ml of Buffer A [25 mM tris(hydroxymethyl)aminomethane (Tris) (pH 8.0), 400 mM NaCl, 10% glycerol] containing 100 μg/ml lysozyme and 3 μg/ml DNaseI. The suspension was rocked for 30 min at 4°C prior to lysis by sonication on ice using a 30-s interval program at an amplitude of 5 for a total of 5 min. Samples were then ultracentrifuged at 39,191 × *g* for 30 min at 4°C. The resulting supernatant was added to 1.5 ml of washed Ni-NTA agarose resin (Qiagen) with 2 mM added imidazole and rocked at 4°C for 30 min. The resin was then washed with 30 ml of wash buffer (30 mM imidazole in Buffer A) and His_6_-SUMO-HtpG was eluted with 10 ml of elution buffer (200 mM imidazole in Buffer A). The eluate fractions were dialyzed against 2 L of Buffer A overnight using a 10-kDa MWCO Slide-A-Lyzer dialysis cassette (Pierce). His_6_-SUMO protease (0.04 mg/ml; His-Ulp1, purified from Addgene #31122) was added to the dialysis sample to cleave the His_6_-SUMO tag from each protein as has been previously described (Uehara et al., 2010). To separate His-tagged and non-tagged proteins, the dialysis samples were then incubated with 1.5 ml of washed Ni-NTA resin for 1 h at 4°C. The flow-through and 3 ml of Buffer A were passed over the column, collected, and contained the desired untagged protein without non-native residues. The protein sample was concentrated to <1 ml using a 30-kDa MWCO Amicon Ultra Centrifugal Filter Device (Millipore) at 4°C followed by flash freezing with N_2__(_*_*l*_*_)_ and storage at −80°C.

Differential scanning fluorimetry was carried out as previously described (Niesen et al., 2007). Briefly, assays were conducted in 25 mM Tris at pH 8.0, 400 mM NaCl, and 5% glycerol. After selecting the optimal protein concentration to be used, HtpG was mixed with test compounds and Sypro dye. The binding of the Sypro dye to HtpG as the temperature increased (25–90°C) was monitored using the Bio-Rad CFX qRT-PCR detection system and melting curves were derived using the CFX Maestro Analysis Software (Bio-Rad Laboratories, United States).

### Statistical Analysis

Comparisons were analyzed by a two-tailed Student’s *t*-test with GraphPad Prism 8. Values of *p* < 0.05 were considered significant. CARA assays and CFU data were presented as means ± standard deviation. Unless otherwise specified, experiments were carried out in triplicate.

## Data Availability Statement

The raw data supporting the conclusions of this article will be made available by the authors, without undue reservation, to any qualified researcher.

## Author Contributions

LL, RS, BG, CN, and JA conceived and designed the study. LL, TL, JR, YL, ZE, XL, and SP performed the experiments. RS, QN, and FS contributed to chemical synthesis. LL, ZE, XL, BG, J-EH, MA, JS, CN, and JA analyzed the data. LL wrote the manuscript. LL, CN, JA, BG, KL, TL, SP, XL, and ZE edited the manuscript. All authors contributed to the article and approved the submitted version.

## Conflict of Interest

The authors declare that the research was conducted in the absence of any commercial or financial relationships that could be construed as a potential conflict of interest.
