# Supplementary material for: Activity-Based Protein Profiling Reveals That Cephalosporins Selectively Active on Non-replicating Mycobacterium tuberculosis Bind Multiple Protein Families and Spare Peptidoglycan Transpeptidases
Source: Front Microbiol. 2020 Jun 23;11:1248. doi: 10.3389/fmicb.2020.01248 (PMC7324553; doi:10.3389/fmicb.2020.01248)
Supplement: Supplementary file 1 [file Data_Sheet_1.PDF]

A.

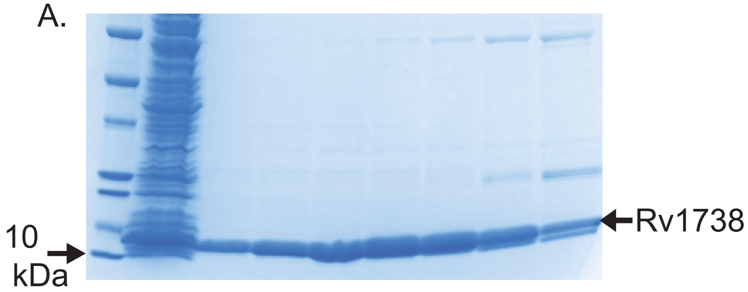

Supplemental Figure S1. Nickel-NTA purification of His_{6x} tagged Rv1738. Coomassie stained gel of Rv1738 (11 kDa) purification.

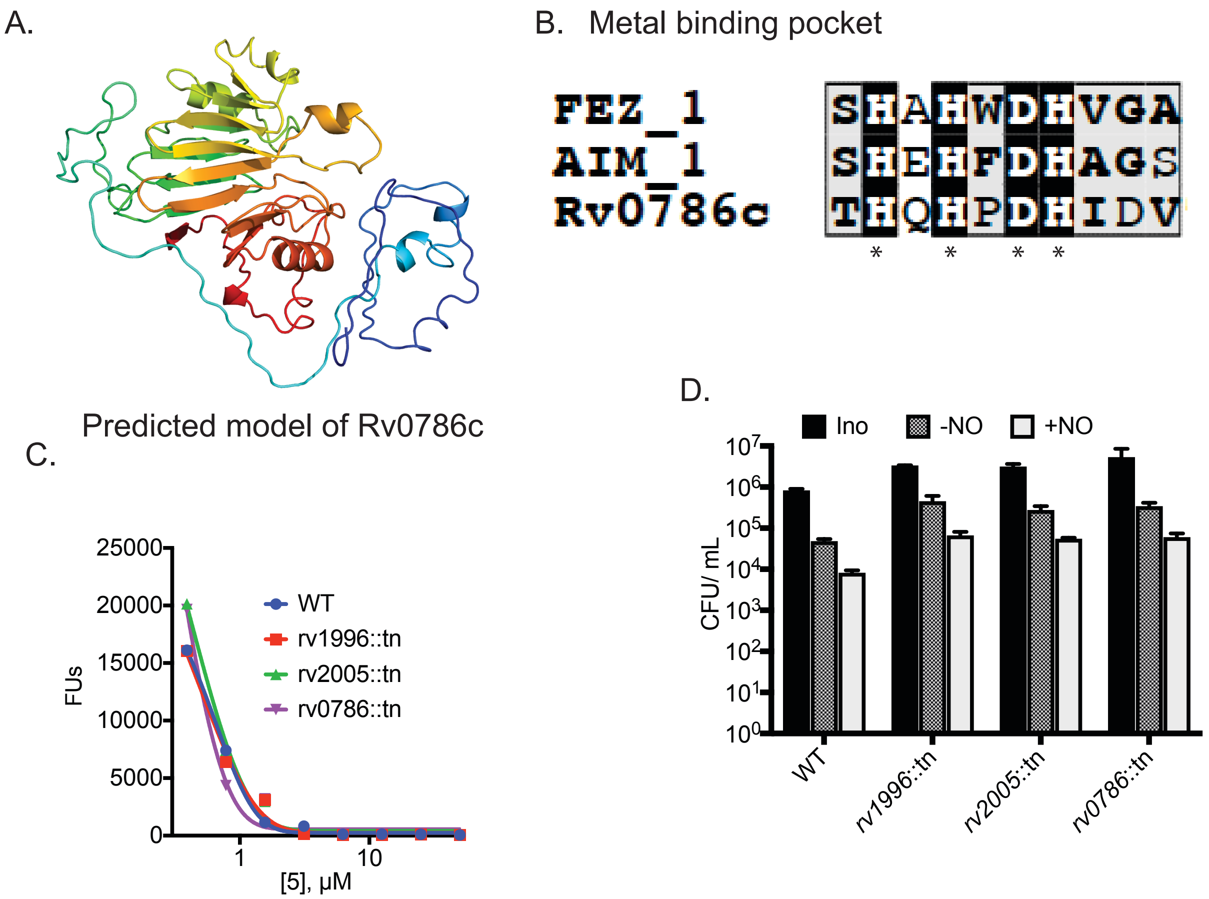

Supplemental Figure S2. Characterization of potential NR-cephalosporin binding partners: Rv1996, Rv2005, and Rv0786. **A.** Predicted 3D structure of Rv0786 using Phyre^2 modelling software. **B.** ClustalW to align the protein sequences of class B3 metallo-β-lactamases FEZ-1 and AIM-1, shown are the principle residues of the metal binding sites. **C.** Activity of **5** against predicted loss of function transposon mutants of *rv1996*, *rv2005*, and *rv0786*. **D.** Sensitivity of these mutants to the presence of acidified nitrite in NR conditions.

Figure S3

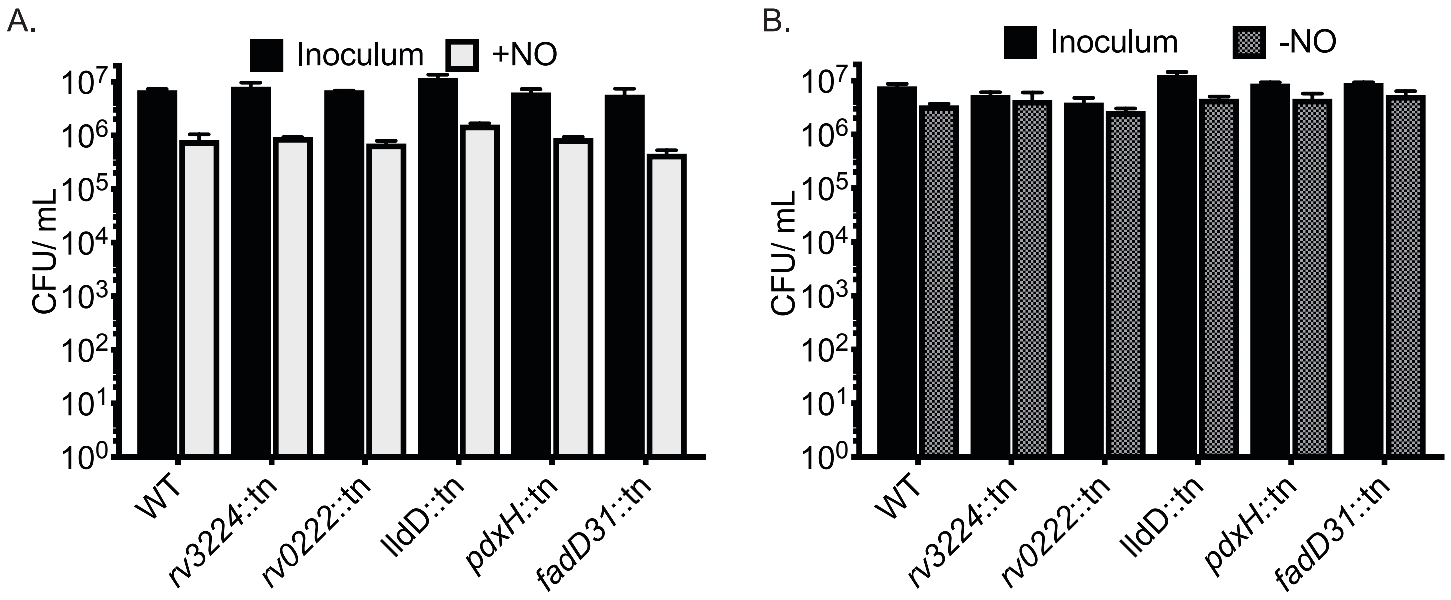

Supplemental Figure S3. Characterization of potential NR-cephalosporin binding partners: Rv3224, Rv0222, PdxH, and FadD31.

A and B. Sensitivity of several transposon mutants to the presence or absence of acidified nitrite to produce nitric oxide (NO) under NR conditions.

A.

Compounds	NR MIC (µM) in carbons sources		
	**Butyrate**	**No Carbon**	**Propionate**
**1**	0.78	12.5	0.78
**5**	3.1	>50	3.1
Rif	6.25	12.5	3.1

B.

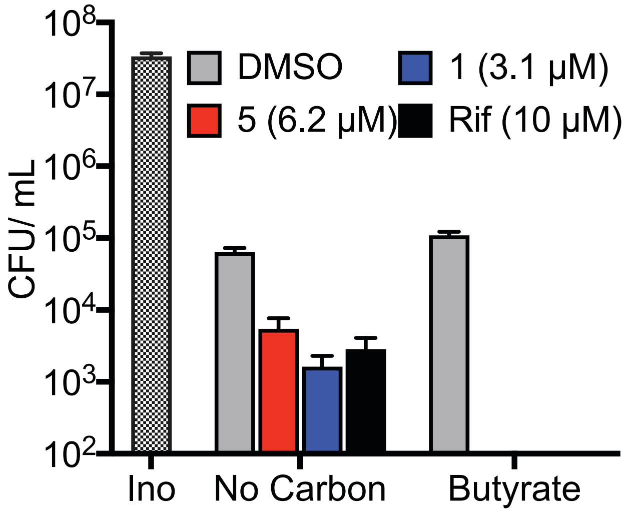

Supplemental Figure S4. Effects of alternative carbon sources on the activity of **1** and **5**. **A.** NR MICs of compounds **1**, **5**, and rifampicin (Rif) after a 7 day incubation using butyrate, propionate, or no carbon source in the NR media. **B.** Enumeration of the number of Mtb cells remaining after 7 days of NR incubation with **1**, **5**, and Rif using no carbon or butyrate in the media.

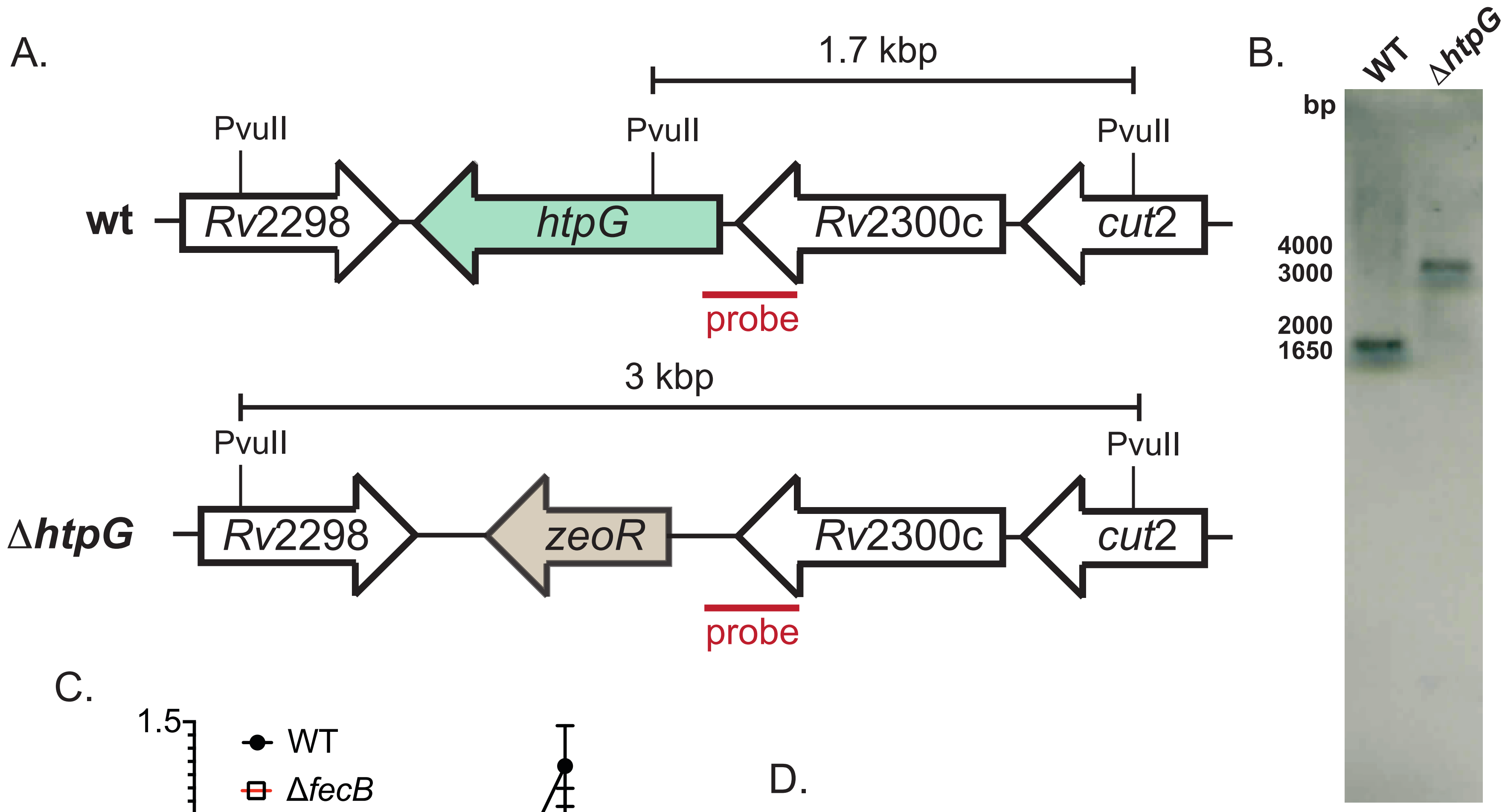

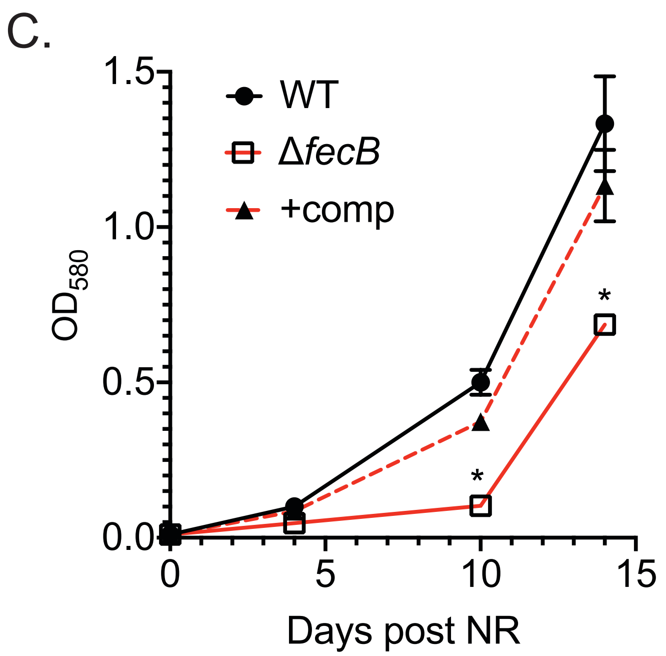

D.

Strains	Compound NR CARA$_{MBC}$, μM	
	**1**	**5**
**WT**	3.1	6.2
ΔhtpG	0.78	1.6
**+comp**	1.6	6.2

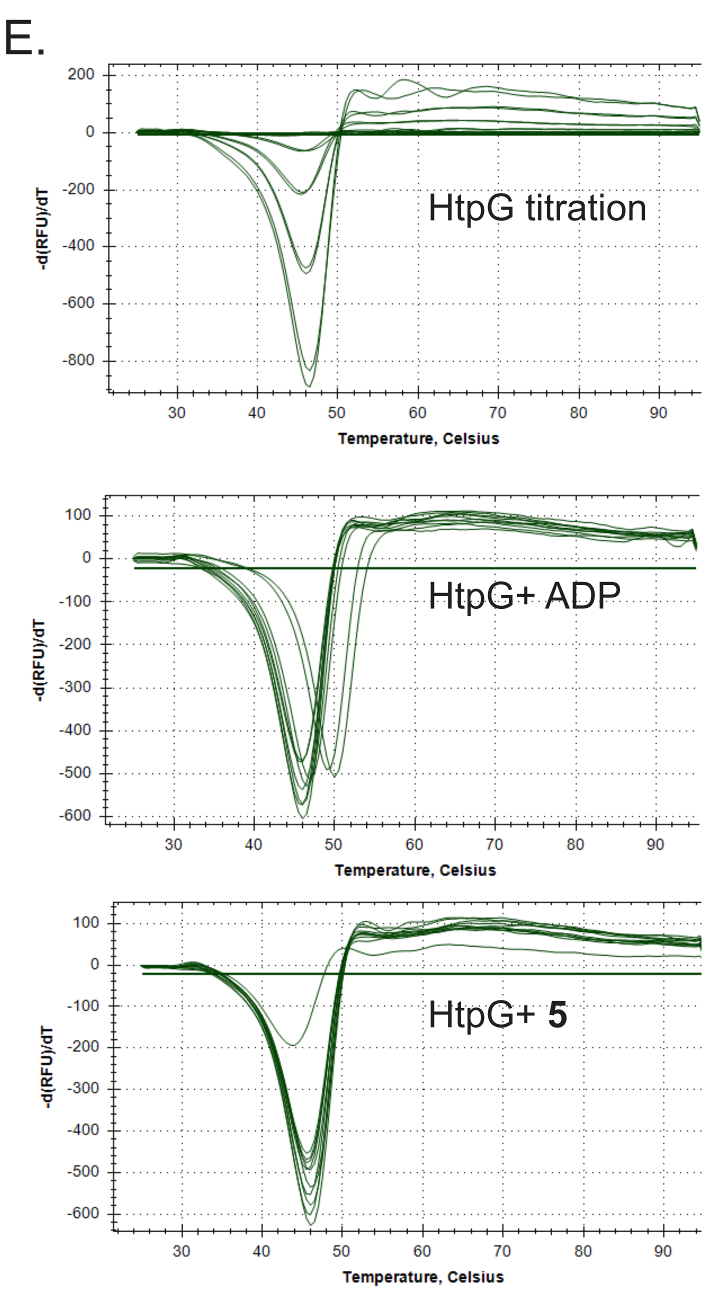

Supplemental Figure S5. Generation of the HtpG knockout and differential scanning fluorimetry of recombinant HtpG. A. Schematic of the htpG gene replacement strategy and the hybridization region of the probe used in Southern blots. B. Southern blot indicating the replacement of htpG with zeoR. C. The recovery of fecB mutant and complemented strain after three days under NR conditions, *$p<0.005$. D. Activity of 1 and 5 against Mtb strains lacking HtpG and the HtpG-reconstituted strain. E. Interaction between 5 and HtpG was assessed by DSF and compared to the binding of known ligand ADP
