# Supplementary material for: Activity-Based Protein Profiling Reveals That Cephalosporins Selectively Active on Non-replicating Mycobacterium tuberculosis Bind Multiple Protein Families and Spare Peptidoglycan Transpeptidases
Source: Front Microbiol. 2020 Jun 23;11:1248. doi: 10.3389/fmicb.2020.01248 (PMC7324553; doi:10.3389/fmicb.2020.01248)
Supplement: Supplementary file 2 [file Data_Sheet_2.docx]

| Table S1: | MIC, µM | |
| --- | --- | --- |
| **Compds** | **Non-replicating*** | **Replicating** |
| 1 | 3.1 | >100 |
| C1a | 12.5 | >100 |
| N1 | 3.1 | >100 |
| C5 | 6.25 | >100 |
| N5 | 3.1 | >100 |
| Rif | 0.625 | 0.08 |
| OPB** | 3.1 | NT |
| *Requires a replicating outgrowth phase | | |

**Oxyphenbutizone

| **Table S2: Relative abundance of putative protein binders** | | | | | | |  |
| --- | --- | --- | --- | --- | --- | --- | --- |
| **C5 pulldown proteins: precipitate fraction** | | | | | | |  |
| Rv Num | Gene | **Log_10_(AUC)** | | | | |  |
|  |  | **C5** | **C1a** | **C1a+ 5** | **C1a+ 1** | **C1a at 12.5 uM** |  |
| Rv3224 | - | 6.4 | 8.7 | 8.5 | 7.1 | 6.5 |  |
| Rv1094 | desA2 | 5.7 | 9.0 | 8.8 | 7.8 | 6.5 |  |
| Rv2996c | serA | 5.8 | 8.2 | 7.8 | 6.7 | ND |  |
| Rv1996 | Usp | 5.9 | 8.2 | 8.1 | 6.8 | 5.2 |  |
| Rv3248c | sahH | 5.8 | 8.6 | 8.4 | ND | 6.2 |  |
| Rv1309 | atpG | 5.9 | 8.2 | 8.2 | ND | 5.8 |  |
| Rv1436 | gap | 6.4 | 8.6 | 8.4 | 7.1 | 6.2 |  |
| Rv2299c | htpG | 6.1 | 8.3 | 7.9 | 6.8 | 5.0 |  |
| Rv2005c | Usp | 6.0 | 8.6 | 8.4 | ND | ND |  |
| Rv3001c | ilvC | 6.5 | 8.6 | 8.4 | 7.7 | 6.5 |  |
| Rv0651 | rplJ | 6.2 | 8.3 | 8.3 | 7.3 | ND |  |
| Rv3457c | rpoA | 6.1 | 8.4 | 8.2 | 6.9 | 5.8 |  |
| Rv0896 | gtlA2 | 6.7 | 8.9 | 8.7 | 7.3 | 6.8 |  |
| Rv2889c | tsf | 6.0 | 7.9 | 7.8 | ND | 5.8 |  |
| Rv1925 | fadD31 | 5.8 | 8.4 | 7.9 | 6.9 | 5.8 |  |
| Rv2623 | Usp | 6.7 | 9.0 | 8.0 | 7.0 | 9.1 |  |
| Rv2220 | glnA1 | 6.2 | 8.5 | 7.5 | 6.3 | 8.8 |  |
| Rv1308 | atpA | 6.2 | 8.4 | 7.0 | 6.5 | 8.8 |  |
| Rv1310 | atpD | 6.5 | 8.8 | 7.5 | 6.8 | 9.1 |  |
| Rv0685 | tuf | 6.2 | 8.4 | 7.3 | 6.3 | 8.6 |  |
| Rv0694 | lldD | 6.5 | 8.4 | 7.5 | 6.3 | 8.6 |  |
| Rv0685 | desA1 | 6.6 | 8.8 | 7.8 | 7.0 | 9.0 |  |
| **C5 pulldown proteins: soluble fraction** | | | | | | |  |
|  |  | **C5** | **C1a** | **C1a+ 5** | **C1a+ 1** | **C1a at low conc** | **C5 at low conc** |
| Rv0222 | echA1 | 5.9 | 5.8 | 5.6 | ND | ND | 6.8 |
| Rv3044 | fecB | 6.0 | ND | 6.3 | ND | 6.2 | 6.9 |
| Rv0250c | - | ND | ND | ND | ND | ND | 7.4 |
| Rv2244 | AcpM | ND | 6.0 | 6.0 | ND | 5.4 | 7.2 |
| Rv1738 | - | ND | ND | ND | ND | 6.5 | 7.5 |
| Rv2607 | pdxH | 6.1 | 6.3 | 6.0 | ND | ND | ND |
| Rv0786c | - | 5.8 | ND | ND | ND | ND | 7.4 |
| Rv2889c | tsf | 6.7 | 6.4 | ND | ND | 5.9 | 7.5 |
| Rv3389c | htdY | 6.5 | 5.7 | ND | ND | ND | 7.6 |

ND: Not detected

| **Table S3: Primers used in this study** | | |
| --- | --- | --- |
| No. | Primer name | Primer sequence |
| 1 | KO-htpGtb-attB1 | GGGGACTGCTTTTTTGTACAAACTTGCTAAATCCGCAGCTTGTCTAGCGCATCGGAGGCATTCG |
| 2 | KO-htpGtb-attB2 | GGGGACAGCTTTCTTGTACAAAGTGGCACCAGGACCGCGCAGACGATGCCGAGAA |
| 3 | KO-htpGtb-attB3 | GGGGACAACTTTGTATAATAAAGTTGTGGGCAAAAGCGAGCGTATTCTCGGGGAGGCCCTC |
| 4 | KO-htpGtb-attB4 | GGGGACAACTTTGTATAGAAAAGTTGGGATTGGTGCTGGTGGACACCGGCTTCGGTATCC |
| 5 | upstream_KOhtpG_fow | CACCGCTGGGTTTTGCACTG |
| 6 | upstream_KOhtpG_rev | CCGCAGAAACGCGTCCTTATTG |
| 7 | SUMO_htpG_fow | GAGGCTCACAGAGAACAGATTGGTGGG ATGAACGCCCATGTCGAGCAG |
| 8 | SUMO_htpG_rev | CTTTCGGGCTTTGTTAGCAGCCGGATCAG CTACAAGGTACGCGCGAGAC |
| 9 | clo-zeoR-lox2-attB1 | GGGGACAAGTTTGTACAAAAAAGCAGGCTATAACTTCGTATAATGTATGCTATACGAAGTTATTGTTGACAATTAATCATCGGCATAG |
| 10 | clo-zeoR-lox2-attB2r | GGGGACCACTTTGTACAAGAAAGCTGGGTATAACTTCGTATAGCATACATTATACGAAGTTATCGCAACGTTCAAATCCGCTCCCGGCG |
| 11 | attB2r_SD_linker_htpG_fow | GGGGACAGCTTTCTTGTACAAAGTGGCTAAGGAGGTATCTCCIATGAACGCCCATGTCGAGCAGT |
| 12 | attB3_htpG_rev | GGGGACAACTTTGTATAATAAAGTTGCTACAAGGTACGCGCGAGACGTTC |
